# Changes in the Mucosa-Associated Microbiome and Transcriptome across Gut Segments Are Associated with Obesity in a Metabolic Syndrome Porcine Model

**DOI:** 10.1128/spectrum.00717-22

**Published:** 2022-07-07

**Authors:** Song-Song Xu, Nan Wang, Lei Huang, Xiu-Ling Zhang, Shu-Tang Feng, Sha-Sha Liu, Yue Wang, Zhi-Guo Liu, Bing-Yuan Wang, Tian-Wen Wu, Yu-Lian Mu, Shao-Hua Hou, Kui Li

**Affiliations:** a Shenzhen Branch, Guangdong Laboratory of Lingnan Modern Agriculture, Genome Analysis Laboratory of the Ministry of Agriculture and Rural Affairs, Agricultural Genomics Institute at Shenzhen, Chinese Academy of Agricultural Sciences, Shenzhen, China; b State Key Laboratory of Animal Nutrition, Key Laboratory of Animal Genetics Breeding and Reproduction of Ministry of Agriculture and Rural Affairs of China, Institute of Animal Sciences, Chinese Academy of Agricultural Sciences, Beijing, China; c College of Animal Science and Technology, Nanjing Agricultural University, Nanjing, China; Huazhong University of Science and Technology

**Keywords:** obesity, mucosa-associated microbiota, 16S rRNA gene sequencing, transcriptome, Inflammatory responses

## Abstract

Several studies have suggested a role for gut mucosa-associated microbiota in the development of obesity, but the mechanisms involved are poorly defined. Here, the impact of the gut mucosa-associated microbiota on obesity and related metabolic disorders was evaluated in a metabolic syndrome (MetS) porcine model. Body composition was determined among male Wuzhishan minipigs consuming a high-energy diet (HED) and compared to that of those consuming a normal diet (ND), and gut segments (duodenum, jejunum, ileum, cecum, colon, and rectum) were sampled for paired analysis of mucosa-associated microbiota and transcriptome signatures with 16S rRNA gene and RNA sequencing, respectively. Our data indicated that long-term HED feeding significantly increased body weight and visceral fat deposition and aggravated metabolic disorders. Specially, HED feeding induced mucosa-associated microbiota dysbiosis and selectively increased the abundance of the families *Enterobacteriaceae*, *Moraxellaceae*, and *Lachnospiraceae* in the upper intestine. The association analysis indicated that specific bacteria play key roles in adiposity, e.g., Lactobacillus johnsonii in the duodenum, Actinobacillus indolicus in the jejunum, Acinetobacter johnsonii in the ileum, Clostridium butyricum in the cecum, Haemophilus parasuis in the colon, and bacterium NLAEzlP808, *Halomonas taeheungii*, and *Shewanella* sp. JNUH029 in the rectum. Transcriptome data further revealed intestinal lipid metabolism and immune dysfunction in the MetS individuals, which may be associated with obesity and related metabolic disorders. Our results indicated that gut mucosa-associated microbiota dysbiosis has the potential to exacerbate obesity, partially through modulating systemic inflammatory responses.

**IMPORTANCE** Obesity is a major risk factor for metabolic syndrome, which is the most common cause of death worldwide, especially in developed countries. The link between obesity and gut mucosa-associated microbiota is unclear due to challenges associated with the collection of intestinal samples from humans. The current report provides the first insight into obesity-microbiome-gut immunity connections in a metabolic syndrome (MetS) porcine model. The present results show that dysbiosis of mucosal microbiota along the entire digestive tract play a critical role in the proinflammatory response in the host-microbial metabolism axis, resulting in obesity and related metabolic disorders in the MetS model.

## INTRODUCTION

Obesity has been identified as a modifiable risk factor for death and loss of productive life years worldwide (1). Specifically, excessive body weight and visceral lipid accumulation have been recognized as major characteristics of obesity which further contribute to disturbed glucose and lipid metabolism (2). Increasing evidence supports the role of the gut microbiota as a crucial player in the pathogenesis of diet-induced obesity and related metabolic complications (3, 4). Development of obesity has been associated with specific microorganisms and metabolites that lead to inflammatory and immune reactions in the intestine (4). For example, obese patients consuming a high level of processed and animal-derived foods have an increased abundance of *Erysipelotrichaceae*, *Ruminococcaceae* species of the *Blautia* genus, and Streptococcus species (5). However, the underlying obesity-microbiome-gut immunity interactions remain largely unknown due to the challenges associated with such studies, such as the ethical and logistical constraints involved in obtaining human intestinal tissues.

The bacterial community shows a distinct distribution along the mammalian gastrointestinal (GI) tract, both longitudinally (proximal to distal) and radially (mucosa to lumen) (6). However, few studies have analyzed microbiota profiles in the gut proximal regions or those living within the outer mucosal layer, which may be dissimilar to the fecal microbiota. The microbiota colonizing the outer mucosal layer, which can interface with the epithelial layer, may play a pivotal role in GI immune cell composition (7). A recent study showed that gut mucosa-associated microbes including *Bacteroidetes* and *Erysipelotrichaceae* in nonobese diabetic mice could be used as biomarkers for type 1 diabetes development (8). Thus, characterization of the gut mucosal microbial community may contribute to our understanding of host-microbiome interactions in both healthy and disease states.

Moreover, long-term dietary interventions can provide a constant source of substrates to continuously shape the gut ecosystem (5). For example, the long-term consumption of a high-energy diet (HED) has been extensively studied as a major cause of obesity and related metabolic diseases such as diabetes (types 1 and 2), Crohn’s disease, ulcerative colitis, liver cirrhosis, and atherosclerosis (9–12). However, the long-term effects of specific diets in humans remain largely unknown due to challenges such as controlling actual nutrient intake (13). Identification of appropriate animal models would therefore aid in understanding the potential impact of dietary effects on the gut microbiome. Some studies have used a porcine model to investigate these interactions, because pigs and humans have highly similar physiological activities, such as gut microbiome colonization and metabolic and immune functions (14, 15). A previous study revealed that an intergenerational pig model of dietary restriction provided an opportunity to understand which features in the developing pig microbiome were causally linked to regulation of various growth parameters (16). Recently, a study of the metabolic syndrome (MetS) porcine model by our group indicated that a long-term HED altered the microbiome of gut contents, decreasing levels of butyrate-producing bacteria, including the genus *Bacteroides* and the families *Lachnospiraceae* and *Ruminococcaceae* (17).

This study was conducted in the MetS porcine model to investigate alterations to the gut mucosa-associated microbiota and their effects on obesity and related metabolic disorders. Here, we reported changes in the gut mucosa-associated microbiota and intestinal transcriptome in different segments of the GI tract and the correlation of microbiota with metabolic parameters (body weight; liver, heart, and spleen weight; visceral lipid accumulation; serum cholesterol levels). This study identified specific gut mucosa-associated microbes that may influence obesity and related metabolic disorders.

## RESULTS

### Long-term HED exacerbates obesity and related metabolic disorders in the MetS model.

After 64 months of dietary interventions, fat accumulation in the viscera (liver, heart, and spleen) was significantly greater (*P < *0.05) in the HED feeding group than in the ND feeding group (Table 1). Coupled with our previous report (17), these results demonstrated that long-term HED feeding aggravated visceral fat accumulation, serum lipid profiles, and systemic inflammation in the MetS porcine model.

**TABLE 1:** Metabolic parameters by diet group

Metabolic parameter^*a*^	ND feeding group (mean ± SEM)	HED feeding group (mean ± SEM)^*b*^	*P* value^*c*^
Body wt (kg)	50.44 ± 4.18B	78.49 ± 5.35A	0.002
Perirenal fat (g)	75.98 ± 10.92B	142.61 ± 19.37A	0.022
Omentum (g)	52.75 ± 7.33B	177.42 ± 32.91A	0.010
Leaf fat (g)	200.02 ± 45.33B	966.24 ± 227.57A	0.018
Liver (g)	656.67 ± 73.24B	970.00 ± 83.52A	0.021
Heart (g)	177.77 ± 13.41B	236.58 ± 15.31A	0.018
Spleen (g)	67.27 ± 11.88B	153.64 ± 20.38A	0.007
TC (mmol/l)	1.79 ± 0.15B	3.63 ± 0.27A	0.001
HDL (mmol/l)	1.04 ± 0.06B	1.94 ± 0.15A	0.002
LDL (mmol/l)	1.30 ± 0.10B	2.44 ± 0.21A	0.004
TGs (mmol/l)	0.32 ± 0.03	0.37 ± 0.07	0.653

a TC, total cholesterol; HDL, high-density lipoprotein; LDL, low-density lipoprotein; TGs, triglycerides.

b Capital letters indicate statistically significant differences between the HED and ND groups (*P *< 0.05). SEM, standard error of the mean.

c *P* values were calculated with Student’s *t* test (*n* = 6 to 9 per group). HED, high-energy diet; ND, normal diet.

### Gut mucosa-associated microbial diversity was decreased across different gut segments in the MetS model.

**FIG 1:**
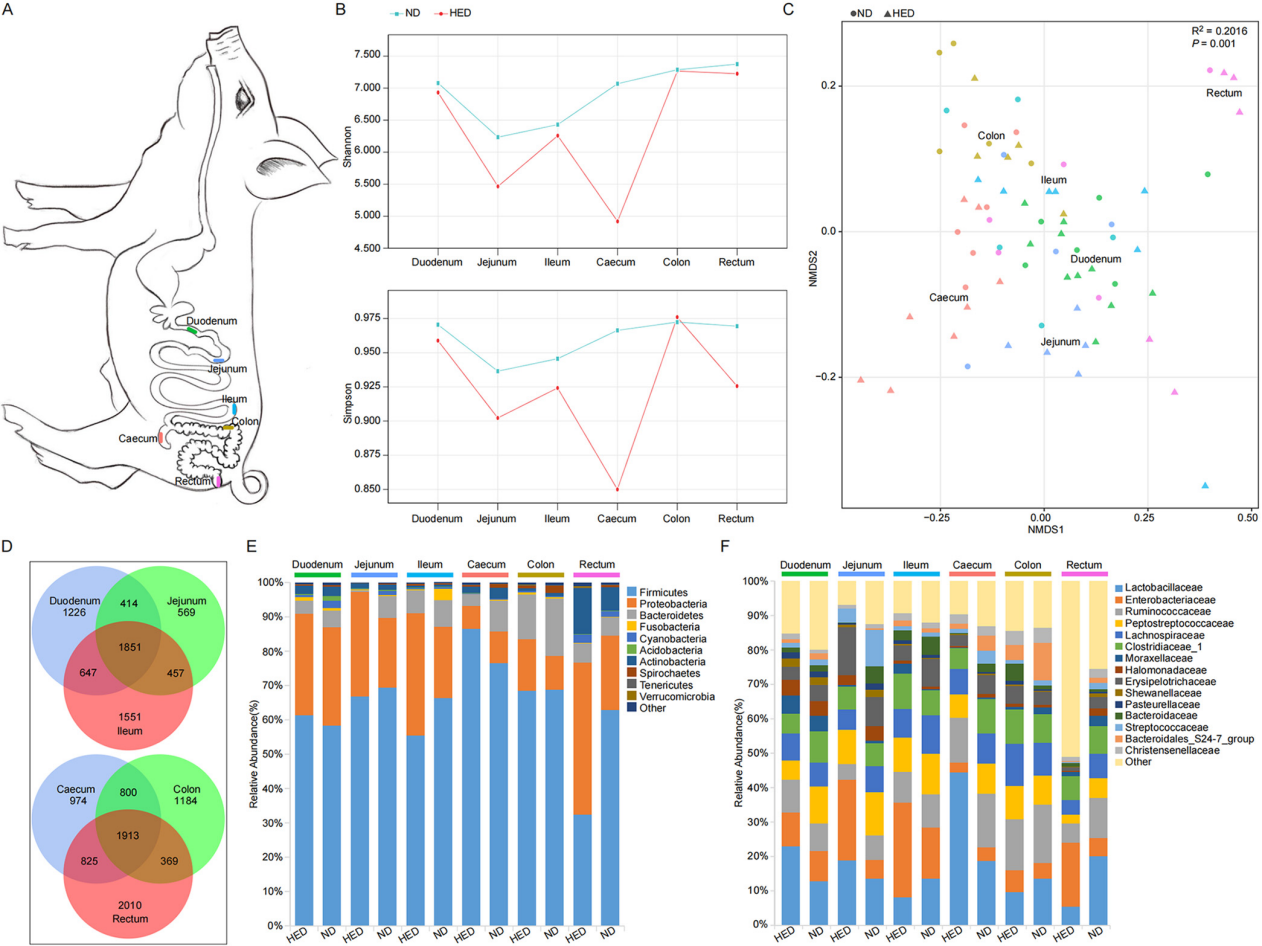
Mucosa-associated microbiome analysis of the metabolic syndrome porcine model with 16S rRNA gene sequencing. (A) Outline of the pig gastrointestinal tract with delineation of gut segments sampled for analyses. (B) The mucosa-associated microbial alpha diversity analysis at the OTU level, illustrated by Shannon and Simpson indices. (C) Beta diversity was evaluated with nonmetric multidimensional scaling (NMDS) using Bray-Curtis dissimilarity. (D) Venn diagrams showing shared OTUs in the small and large intestine, respectively. (E and F) Taxonomic summary of top 10 phyla (E) and top 15 families (F) (by abundance), represented by mean values per group and gut section. HED, high-energy diet; ND, normal diet.

We sequenced a total of 92 intestinal mucosal specimens along the entire digestive tract (including duodenum, jejunum, ileum, cecum, colon, and rectum), using bacterial 16S rRNA gene sequencing, in the HED group (*n* = 11) and the ND group (*n* = 6). The mucosa-associated microbial diversity was analyzed in the six individual gut segments (Fig. 1A). Rarefaction curves revealed that the estimated operational taxonomic unit (OTU) richness nearly reached saturation in each region of the GI tract (see Fig. S1A in the supplemental material). Generally, the HED feeding group had lower alpha (within-sample) diversity at the OTU level than the ND feeding group as measured by the Shannon and Simpson indices (Fig. 1B). We calculated the bacterial copy number per gram of gut mucosa-associated contents using quantitative real-time PCR (qPCR) in the duodenum, jejunum, ileum, cecum, and colon. The HED feeding group showed highly significantly (*P < *0.01) lower copy numbers per gram of total bacteria compared to the ND feeding group (see Fig. S1B). Nonmetric multidimensional scaling (NMDS) with taxonomic information showed a clear separation of microbial community structure between the two feeding groups (Fig. 1C; see also Fig. S2 in the supplemental material). As shown in the NMDS plot, the small intestine samples clustered closely together, whereas large intestine samples clustered less closely on NMDS1 (Fig. 1C), indicating more diverse bacterial composition in the large intestine. Furthermore, 27.57% (1,851 OTUs) were shared among the small intestine samples, whereas 23.69% (1,913 OTUs) were shared among the large intestine samples (Fig. 1D).

### Gut mucosa-associated microbiota dysbiosis across different gut segments in the MetS model.

Analysis at the phylum level indicated that the gut mucosa-associated microbiota was dominated by five major phyla among the different gut segments: *Firmicutes*, *Proteobacteria*, *Bacteroidetes*, *Actinobacteria*, and *Fusobacteria*, together constituting up to 93% of the OTUs in each gut segment, on average (Fig. 1E). Notably, levels of the major bacterial phyla *Firmicutes* and *Proteobacteria* were drastically different from the proximal to distal regions of the GI tract in the MetS model (see Fig. S3). In the rectum, MetS animals showed a significant decrease in *Firmicutes* (*P = *0.028) and increase in *Proteobacteria* (*P = *0.009) compared with the ND feeding group. The average bacterial community compositions at the family level are shown in Fig. 1F. We also compared differences in the microbial communities at the family, genus, and species levels across the GI tract between the groups. The HED-fed pigs had increased colonization of several families, including *Lactobacillaceae* in the duodenum and cecum, *Enterobacteriaceae* in the jejunum, *Moraxellaceae* in the ileum, and *Lachnospiraceae* in the colon (Table 2). However, 12 families were significantly enriched in the ND feeding group compared with the HED feeding group (*P < *0.05): *Bacteroidaceae* in the jejunum, *Erysipelotrichaceae* and *Fusobacteriaceae* in the ileum, *Bacteroidales* S247 group and *Streptococcaceae* in the cecum, *Ruminococcaceae*, *Spirochaetaceae*, and *Rikenellaceae* in the colon, and *Lactobacillaceae*, *Ruminococcaceae*, *Lachnospiraceae*, *Erysipelotrichaceae*, and *Halomonadaceae* in the rectum.

**TABLE 2:** Relative abundances of mucosa-associated bacterial families detected in the HED and ND groups

Intestinal segment	Family	Mean % abundance ± SEM^*a*^		
		HED	ND	*P* value^*b*^
Duodenum	*Lactobacillaceae*	22.92 ± 2.16A	12.83 ± 0.75B	0.002
Jejunum	*Enterobacteriaceae*	23.33 ± 2.91A	5.51 ± 1.08B	0.014
	*Bacteroidaceae*	0.20 ± 0.10B	4.93 ± 3.34A	0.027
Ileum	*Erysipelotrichaceae*	4.33 ± 0.65B	7.90 ± 1.27A	0.019
	*Moraxellaceae*	2.83 ± 1.61A	0.20 ± 0.09B	0.028
	*Fusobacteriaceae*	0.30 ± 0.17B	3.20 ± 2.41A	0.042
Caecum	*Lactobacillaceae*	44.44 ± 7.73A	18.73 ± 3.89B	0.020
	*Bacteroidales* S247 group	1.51 ± 0.55B	4.36 ± 1.32A	0.028
	*Streptococcaceae*	1.21 ± 0.62B	3.69 ± 1.06A	0.039
Colon	*Ruminococcaceae*	14.72 ± 0.78B	16.97 ± 0.43A	0.047
	*Lachnospiraceae*	12.35 ± 0.38A	9.51 ± 0.91B	0.016
	*Spirochaetaceae*	0.68 ± 0.09B	2.11 ± 0.35A	0.016
	*Rikenellaceae*	0.80 ± 0.13B	1.69 ± 0.14A	0.009
Rectum	*Lactobacillaceae*	5.38 ± 3.08B	20.00 ± 4.46A	0.016
	*Ruminococcaceae*	5.69 ± 0.77B	11.53 ± 1.83A	0.028
	*Lachnospiraceae*	4.27 ± 0.31B	7.11 ± 0.91A	0.028
	*Erysipelotrichaceae*	0.93 ± 0.11B	3.37 ± 0.83A	0.009
	*Halomonadaceae*	0.45 ± 0.10B	2.03 ± 0.41A	0.028

a Capital letters indicate statistically significant differences between HED and ND feeding groups (*P *< 0.05).

b *P* values were determined by Mann-Whitney *U* test (*n* = 5 to 11 per diet group). HED, high-energy diet; ND, normal diet.

**FIG 2:**
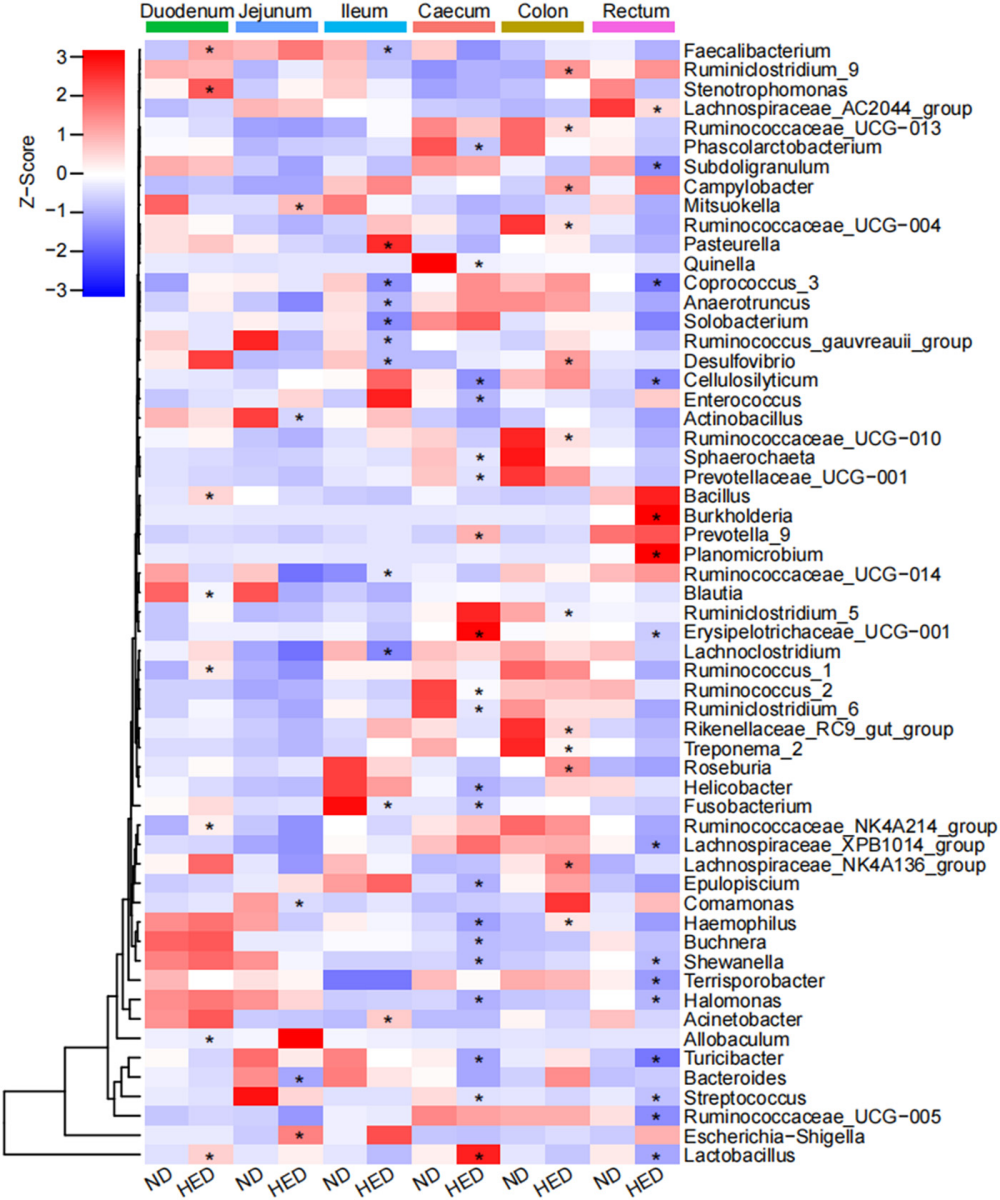
Changes in mucosa-associated microbes at the genus level in different regions of the GI tract in the metabolic syndrome (MetS) porcine model. *, *P < *0.05 (Mann-Whitney *U* test).

At the genus level, only 23 genera were significantly different in small intestinal regions between the treatment groups (*P < *0.05) (Fig. 2). In the duodenal region, the genera *Bacillus*, *Faecalibacterium*, *Lactobacillus*, *Ruminococcaceae* NK4A214 group, *Ruminococcus* 1, and *Stenotrophomonas* were more abundant in the HED feeding group, whereas the genera *Allobaculum* and *Blautia* were less abundant. In the jejunal region, Escherichia*-Shigella* and *Mitsuokella* were more abundant and *Actinobacillus*, *Bacteroides*, and *Comamonas* were less abundant in the HED feeding group. Additionally, Acinetobacter, *Pasteurella*, and *Ruminococcaceae* UCG-014 were more abundant in the ileum region of the HED feeding group than the ND feeding group. However, *Anaerotruncus*, *Coprococcus* 3, *Desulfovibrio*, *Faecalibacterium*, *Fusobacterium*, *Lachnoclostridium*, *Ruminococcus* *gauvreauii* group, and *Solobacterium* were less abundant in the HED feeding group. In contrast to the small intestine, 39 genera were altered across the large intestinal regions. In the cecal luminal region of the HED feeding group, *Erysipelotrichaceae* UCG-001, *Lactobacillus*, and *Prevotella* 9 were significantly more abundant and 17 genera (including *Turicibacter*, Streptococcus, *Ruminococcus* 2, *Ruminiclostridium* 6, and *Halomonas*) were less abundant in the HED feeding group (*P < *0.05). In the colonic regions, *Lachnospiraceae* NK4A136 group, *Roseburia*, Haemophilus, *Desulfovibrio*, Campylobacter, and *Ruminiclostridium* 9 were significantly more abundant in the HED feeding group than in the ND feeding group (*P < *0.05). However, *Rikenellaceae* RC9 gut group, Treponema 2, *Ruminococcaceae* UCG-010, *Ruminiclostridium* 5, *Ruminococcaceae* UCG-013, and *Ruminococcaceae* UCG-004 were less abundant in the HED feeding group. In the rectal region of the HED feeding group, *Planomicrobium* and *Burkholderia* were more abundant (*P < *0.05) than in the ND feeding group. Additionally, 13 genera (including *Lactobacillus*, *Turicibacter*, *Halomonas*, *Terrisporobacter*, and Streptococcus) were less abundant in the HED feeding group than in the ND feeding group.

The two treatment groups also exhibited marked changes in mucosa-associated microbiota composition at the species level across all luminal regions (Table 3). Lactobacillus johnsonii showed the largest difference in abundance in the luminal regions of the duodenum, ileum, and rectum. Interestingly, Lactobacillus johnsonii was most abundant in the duodenal lumen of the HED feeding group (12.60%) and then steadily decreased through the GI tract to the rectal lumen, where it reached 1.9%. Furthermore, several groups were more abundant in the HED feeding group, namely, Stenotrophomonas maltophilia in the duodenum, Shigella flexneri and Shigella sonnei in the jejunum, Acinetobacter johnsonii and bacterium NLAE-zl-P808 in the ileum, Lactobacillus reuteri in the cecum, Haemophilus parasuis and Actinobacillus porcinus in the colon, and bacterium NLAE-zl-P808, *Enterobacteriaceae* bacterium_X2/SB59, Klebsiella oxytoca, *Pantoea* sp. FSGSA15, *Planococcus* sp. BM-G6, Planomicrobium okeanokoites, and Pseudomonas putida in the rectum. Collectively, these results indicated that the long-term HED modulated the mucosa-associated microbial community structure, resulting in a bacterial composition dissimilar from that of the ND group.

**TABLE 3:** Relative abundances of mucosa-associated bacterial species detected in the HED and ND groups

Intestinal segment	Species	Mean % abundance ± SEM^*a*^		*P* value^*b*^
		HED	ND	
Duodenum	Lactobacillus johnsonii	0.126 ± 0.011A	0.068 ± 0.007B	0.020
	Stenotrophomonas maltophilia	0.003 ± 0.0005A	0.001 ± 0.0005B	0.020
Jejunum	Shigella flexneri	0.182 ± 0.036A	0.033 ± 0.009B	0.014
	Shigella sonnei	0.007 ± 0.003A	0.001 ± 0.0006B	0.014
	Actinobacillus indolicus	0.0003 ± 0.00003B	0.002 ± 0.001A	0.014
	*Actinobacillus* *rossii*	0.0002 ± 0.0001B	0.002 ± 0.0005A	0.014
Ileum	Acinetobacter johnsonii	0.002 ± 0.0006A	0.0004 ± 0.0002B	0.028
	Fusobacterium ulcerans	0.0004 ± 0.0002B	0.002 ± 0.001A	0.034
	Lactobacillus johnsonii	0.040 ± 0.009B	0.083 ± 0.0147A	0.042
	Bacterium NLAE-zl-P808	0.001 ± 0.0002A	0.0006 ± 0.0001B	0.042
Cecum	Shewanella algae	0.002 ± 0.0007B	0.005 ± 0.0006A	0.014
	*Halomonas* sp. Betam1	0.0009 ± 0.0004B	0.003 ± 0.0003A	0.014
	*Helicobacter rappini*	0.0006 ± 0.0005B	0.002 ± 0.0002A	0.020
	Lactobacillus reuteri	0.103 ± 0.025A	0.027 ± 0.007B	0.039
	Clostridium butyricum	0.0005 ± 0.0002B	0.002 ± 0.001A	0.045
Colon	Haemophilus parasuis	0.002 ± 0.0005A	0.0003 ± 0.0002B	0.028
	Actinobacillus porcinus	0.002 ± 0.0004A	0.0005 ± 0.0002B	0.028
Rectum	Bacterium NLAE-zl-P808	0.001 ± 0.0007A	0.00006 ± 0.00002B	0.008
	*Enterobacteriaceae* bacterium X2/SB59	0.003 ± 0.002A	0.0001 ± 0.00009B	0.028
	*Halomonas taeheungii*	0.001 ± 0.0002B	0.005 ± 0.001A	0.028
	Klebsiella oxytoca	0.004 ± 0.0009A	0.001 ± 0.0006B	0.047
	Lactobacillus fermentum	0.0001 ± 0.00004B	0.001 ± 0.0003A	0.016
	Lactobacillus johnsonii	0.019 ± 0.012B	0.114 ± 0.029A	0.016
	*Pantoea* sp. FSGSA15	0.006 ± 0.005A	0.0002 ± 0.0002B	0.035
	*Planococcus* sp. BM-G6	0.001 ± 0.0008A	0.00005 ± 0.00005B	0.031
	Planomicrobium okeanokoites	0.003 ± 0.002A	0.0002 ± 0.0002B	0.047
	Pseudomonas putida	0.014 ± 0.012A	0.0005 ± 0.0005B	0.045
	*Shewanella* sp. JNU-H029	0.002 ± 0.0005B	0.010 ± 0.002A	0.009

a Capital letters indicate statistically significant differences between HED and ND groups (*P *< 0.05).

b *P* values were determined by Mann-Whitney *U* test (*n* = 5 to 11 per diet group). HED, high-energy diet; ND, normal diet.

### Correlations between gut mucosa-associated microbiota composition and obesity and related metabolic disorders.

**FIG 3:**
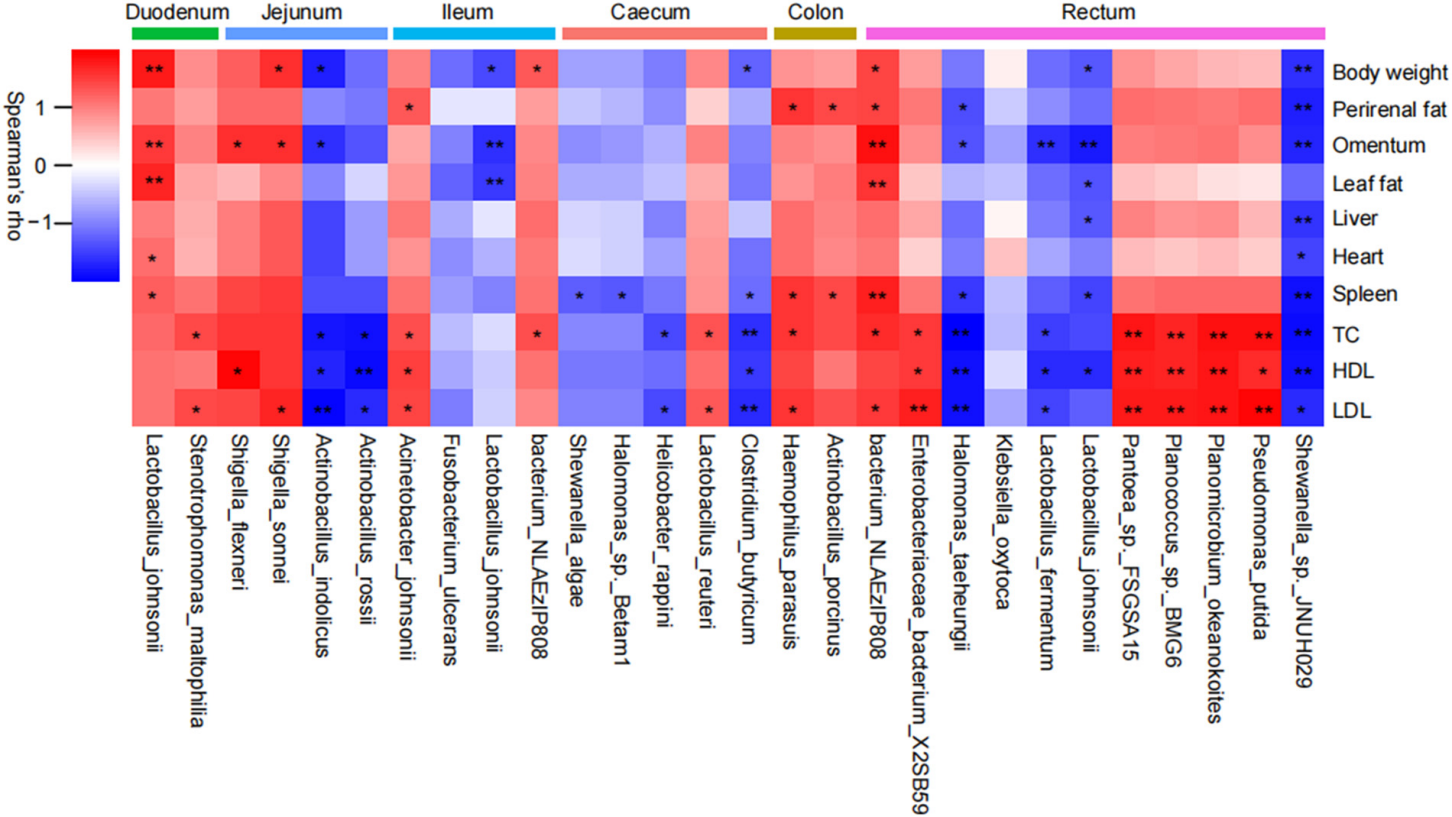
Spearman’s correlation between mucosa-associated microbes and obesity-related metabolic parameters. *, *P < *0.05; **, *P < *0.01 (two-tailed Student's *t* test).

We next focused on the association between mucosa-associated bacterial species and the markers of obesity and related metabolic complications. Analysis based on Spearman’s correlation revealed that the abundance of Lactobacillus johnsonii in the duodenum, Shigella sonnei in the jejunum, Acinetobacter johnsonii in the ileum, Lactobacillus reuteri in the cecum, Haemophilus parasuis in the colon, and bacterium_NLAEzlP808 and *Pantoea* sp. FSGSA15 in the rectum were positively associated with the measured metabolic parameters (Fig. 3). However, Actinobacillus indolicus in the jejunum, Lactobacillus johnsonii in the ileum, Clostridium butyricum in the cecum, and Halomonas taeheungii, Lactobacillus johnsonii, and *Shewanella* sp. JNUH029 in the rectum were negatively associated with obesity and related metabolic disorders (Fig. 3). These results suggested that HED-induced obesity was associated with significant changes in the mucosa-associated microbiota composition.

### Intestinal functions were altered across different gut segments in the MetS model.

We analyzed the RNA sequencing (RNA-seq) data from porcine intestinal tissues, which were generated for each sample of the HED (*n* = 4) and ND (*n* = 4) feeding groups. RNA-seq-based transcriptome profiling revealed a total of 4,889,868,618 raw reads and a total of 4,587,375,920 clean reads after quality control (see Table S1 in the supplemental material). For each sample, the clean reads comprised between 87.10% and 96.72% of the raw reads. Among the clean reads, the percentage of reads with Q30 values ranged from 87.11% to 95.09%, and the average GC content was 45.79%. These results suggested that the RNA-seq data were of good quality.

We identified long noncoding RNAs (lncRNAs) and mRNAs in both the HED and ND feeding groups. The majority of identified lncRNAs were located in intergenic noncoding regions (see Fig. S4A in the supplemental material). The transcript length, exon length, open reading frame (ORF) length, and conservation score of lncRNAs (including both annotated and novel lncRNAs) and mRNAs were compared (see Fig. S4B, C, and D). The novel lncRNAs were found to be significantly shorter in transcript length and ORF length and to have fewer exons than mRNAs, consistent with general characteristics of known lncRNAs. The distributions of exon numbers and ORF lengths showed similar patterns in the annotated and novel lncRNAs. However, the novel lncRNAs were less conserved than protein-coding transcripts, as determined with phastCon (see Fig. S4E); this finding is consistent with a previous report (18).

**FIG 4:**
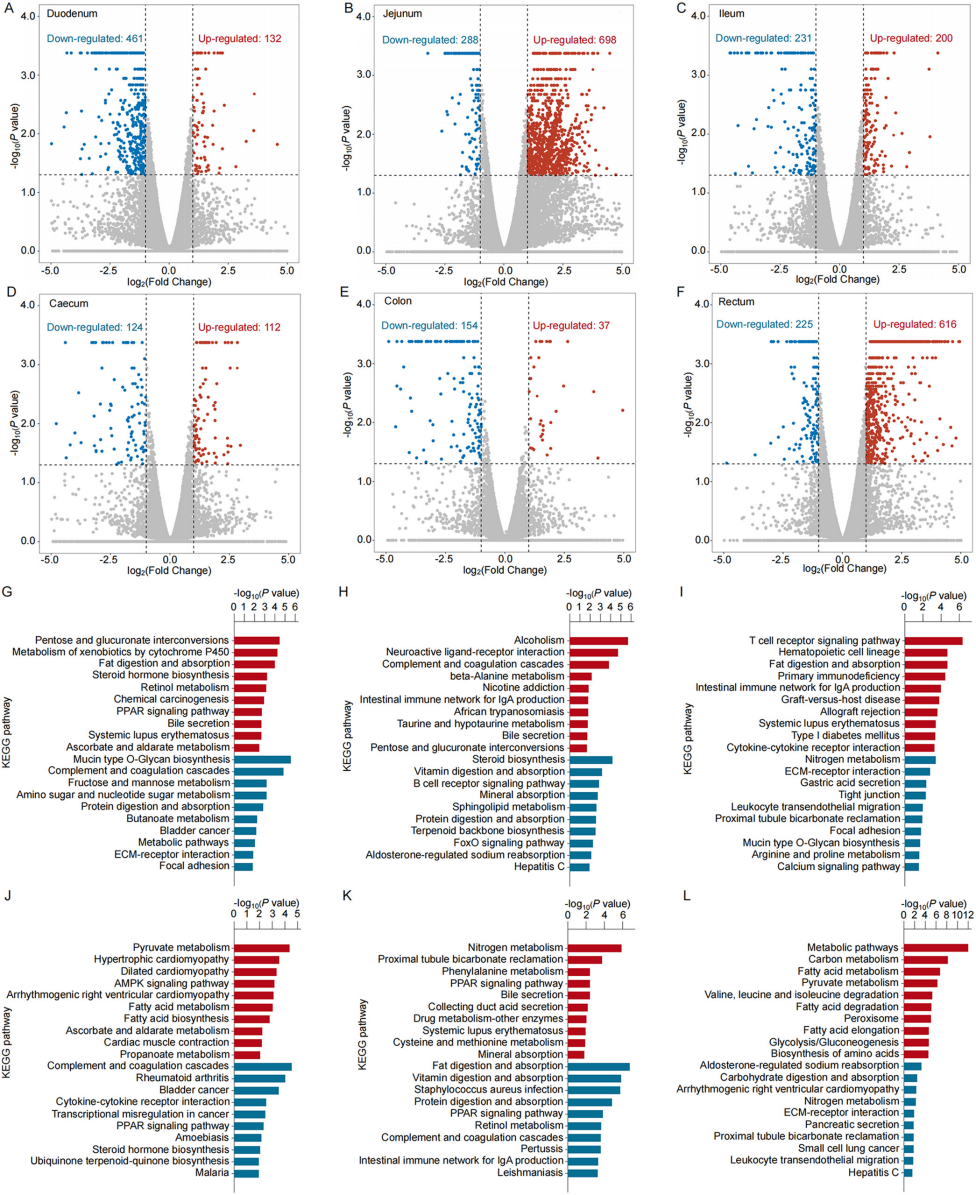
Differentially expressed mRNAs (DEGs) in intestinal tissues of individuals in the HED group compared with those in the ND group. (A to F) The volcano plot shows downregulated (blue), upregulated (red), and unchanged (gray) DEGs in the duodenum (A), jejunum (B), ileum (C), cecum (D), colon (E), and rectum (F). (G to L) Top canonical KEGG pathways in the duodenum (G), jejunum (H), ileum (I), cecum (J), colon (K), and rectum (L). Red represents enriched pathways and blue represents depleted pathways.

We defined differentially expressed genes (DEGs) and differentially expressed lncRNAs (DELs) as those with a false-discovery rate (FDR) of <0.05 and |log_2_(fold change)| of >0.6. Compared to the ND feeding group, there were several hundred DEGs in the small intestine for the HED feeding group; these comprised 132, 698, and 200 upregulated and 461, 288, and 231 downregulated genes in the duodenum, jejunum, and ileum, respectively (Fig. 4A, B, and C; see also Tables S2 to S4 in the supplemental material). There were fewer DEGs in the large intestine for the HED feeding group, including 112, 37, and 616 upregulated and 124, 154, and 225 downregulated genes in the cecum, colon, and rectum, respectively (Fig. 4D, E, and F; see also Tables S5 to S7). Individuals in the HED feeding group displayed significant changes in several biological signaling pathways. In the small intestine, the top pathways associated with the DEGs were related to fat and protein metabolism, bile secretion, pathogen recognition, and inflammatory pathways (Fig. 4G, H, and I). In the large intestine, the top pathways associated with DEGs in the HED feeding group were primarily related to fatty acid, protein, and carbohydrate metabolism and immune system pathways (Fig. 4J, K, and L).

**FIG 5:**
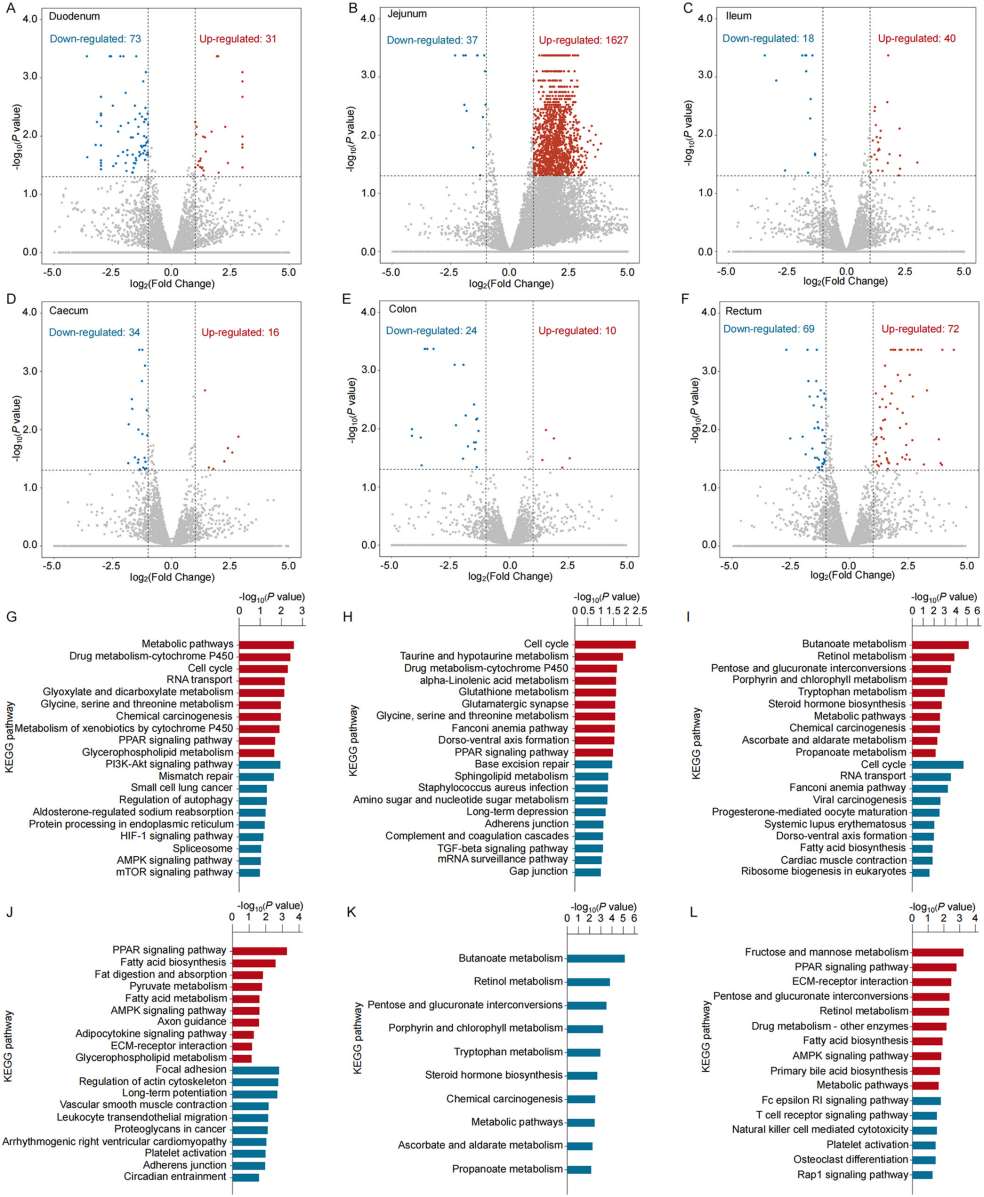
Differentially expressed lncRNAs (DELs) in intestinal tissues of individuals in the HED group compared to those in tissues from the ND group. (A to F) The volcano plot shows downregulated (blue), upregulated (red), and unchanged (gray) DELs in the duodenum (A), jejunum (B), ileum (C), cecum (D), colon (E), and rectum (F). (G to L) Top canonical KEGG pathways of dysregulated mRNAs in coexpressed lncRNA-mRNA pairs with high correlation coefficients in the duodenum (G), jejunum (H), ileum (I), cecum (J), colon (K), and rectum (L). Red represents enriched pathways and blue represents depleted pathways.

We also identified DELs in all of the luminal regions in the HED feeding group: 104 in the duodenum, 1,664 in the jejunum, 58 in the ileum, 50 in the cecum, 34 in the colon, and 141 in the rectum (Fig. 5A to F). To investigate the potential functions of the DELs, we performed lncRNA-mRNA coexpression pair analysis. After filtering, the coexpressed lncRNA-mRNA pairs with high (>0.9) correlation coefficients were selected, including 20 DELs and 1,186 DEGs in the duodenum, 615 and 1,714 in the jejunum, 6 and 1,072 in the ileum, 7 and 110 in the cecum, 4 and 33 in the colon, and 23 and 99 in the rectum (see Tables S8 to S13 in the supplemental material). To understand the main gene functions at a more global level, we functionally annotated the DEGs identified from DELs in the six intestinal segments. The top pathways among all tissues were mainly associated with lipid and steroid metabolism and with immune and inflammatory responses (Fig. 5G to L).

## DISCUSSION

The gut contains a complex and dynamic bacterial community with great potential to influence host health (2, 5). Specifically, the microbiota colonizing the outer mucus layer has critical roles in bacterial-triggered host immune activation and metabolic disorders (15, 19). Thus, the functional characteristics of mucosa-associated microbial communities are key to our understanding of host-microbiome interactions in both healthy and disease states. In our study, all animals were housed in same environmental conditions to ensure that changes in gut microbiota composition could be attributed to diet-specific effects rather than environmental influences. After 64 months of high-energy diet, we successfully established a MetS porcine model characterized by increased body weight, serum lipid, and proinflammatory cytokine levels, visible atheromatous plaque on abdominal aorta, accumulated lipid droplets and enhanced apoptosis in hepatocytes, and impaired intestinal epithelial integrity (17). In our previous study (17), we showed that diet composition impacted gut microbiota structure and that the high-energy diet pattern decreased the abundance of short-chain fatty acid-producing bacteria, including *Bacteroides*, *Lachnospiraceae*, and *Ruminococcaceae*. In this study, we found that the high-energy diet also decreased gut mucosa-associated microbial diversity and disrupted its structure along the entire digestive tract. Notably, the metabolic parameters were strongly correlated with the abundance of gut mucosa-associated microbiota in the MetS model. Coupled with gut transcriptome analysis, these findings indicated that gut mucosa-associated microbiota dysbiosis might exacerbate obesity and related metabolic disorders in the MetS model.

Recently, several studies have implicated mucosa-associated microbiota dysbiosis in the pathogenesis of obesity and related metabolic disorders. A study of the duodenal mucosal microbiota of patients with intestinal metaplasia showed an inverse relationship between microbial diversity and metabolic diseases (20). Another study suggested that patients with morbid obesity had lower jejunal mucosa-associated microbial diversity than healthy controls (21). We here confirmed previous reported results demonstrating that HED-induced obese minipigs had decreased mucosa-associated microbial diversity and richness in the GI tract. Our results showed that the mucosa-associated microbiota along the longitudinal axis of the small and large intestine was dominated by *Firmicutes* and *Proteobacteria*. The abundance level of *Firmicutes* changed drastically along the length of the intestine, reaching the lowest levels in the rectal lumen of the HED feeding group. In contrast, *Proteobacteria* were presented at relatively low levels in the cecum and colon, steadily increased in abundance along the length of the large intestine, and reached the highest levels in the rectum. Interestingly, reduced abundance of *Firmicutes* and high prevalence of *Proteobacteria* have previously been linked to high-polysaccharide diets and lipopolysaccharide production in humans and other mammals (13, 19, 22).

We found that specific *Firmicutes* and *Proteobacteria* were significantly affected by dietary interventions across the GI tract. Consistent with a previous report showing greater *Lactobacillaceae* abundance in Heligmosomoides polygyrus-infected C57BL/6 mice (23), we observed elevated levels of *Lactobacillus* and Lactobacillus johnsonii in the duodenum of HED-fed animals. Lactic acid bacteria are representative probiotics that have demonstrated beneficial effects, such as improvement in epithelial barrier function, immunity enhancement, and anti-inflammatory activities (24). Interestingly, a higher number of *Lactobacillaceae* species (Lactobacillus johnsonii and Lactobacillus reuteri) colonized the upper intestine (duodenum and cecum) of the HED feeding group, which led to mucosal protection and anti-inflammatory effects (5). However, *Lactobacillaceae* species (Lactobacillus johnsonii and Lactobacillus fermentum) were less abundant in the rectal region of the HED feeding group individuals, consistent with an increase in proinflammatory metabolites. Additionally, the HED feeding group showed elevated levels of both jejunal *Enterobacteriaceae* (Escherichia*-Shigella*, Shigella flexneri, and Shigella sonnei) and ileal *Moraxellaceae* (Acinetobacter and Acinetobacter johnsonii) compared with the ND feeding group. *Enterobacteriaceae* is a family of Gram-negative bacteria that has previously been linked to obesity and hepatic damage (25). *Moraxellaceae*, a family of *Proteobacteria*, colonizes mucosal membranes and is associated with hepatic steatosis (26). Moreover, the abundance of the *Halomonadaceae* family (*Halomonas* and Halomonas taeheungii) was lower in the HED feeding group, a result that was also observed in nursery pigs on a high-fat diet (27). Our findings suggest that the mucosa-associated microbiota serves as a link between diet and disease risk by modulating pro- and anti-inflammatory responses.

Previous studies have reported that obesity was associated with chronic systemic inflammation (28). In the current study, Lactobacillus johnsonii in the duodenal region showed significant positive correlations with obesity and associated metabolic disorders, which was likely related to anti-inflammatory responses. However, we found that Lactobacillus johnsonii was inversely correlated with metabolic parameters in the cecal and rectal luminal regions of the HED feeding group. Lactobacillus johnsonii has been shown to have an antiobesity effect by preventing inflammation and mucosal barrier disruption in the gut (29). Moreover, anti-inflammatory-associated bacteria, including jejunal Actinobacillus indolicus, cecal Clostridium butyricum, and rectal Halomonas taeheungii and *Shewanella* sp. JNUH029, were negatively correlated with visceral fat deposition and serum lipid levels, indicating stimulation of proinflammatory metabolites following long-term HED feeding. Further, inflammatory bowel disease-associated bacteria, including ileal Acinetobacter johnsonii, colonic Haemophilus parasuis, and rectal bacterium NLAEzlP808, were positively correlated with visceral fat levels. Consequently, in the MetS model, enrichment of intestinal mucosa-associated pathogenic bacteria induced systemic inflammation, which was related to obesity and related metabolic disorders.

**FIG 6:**
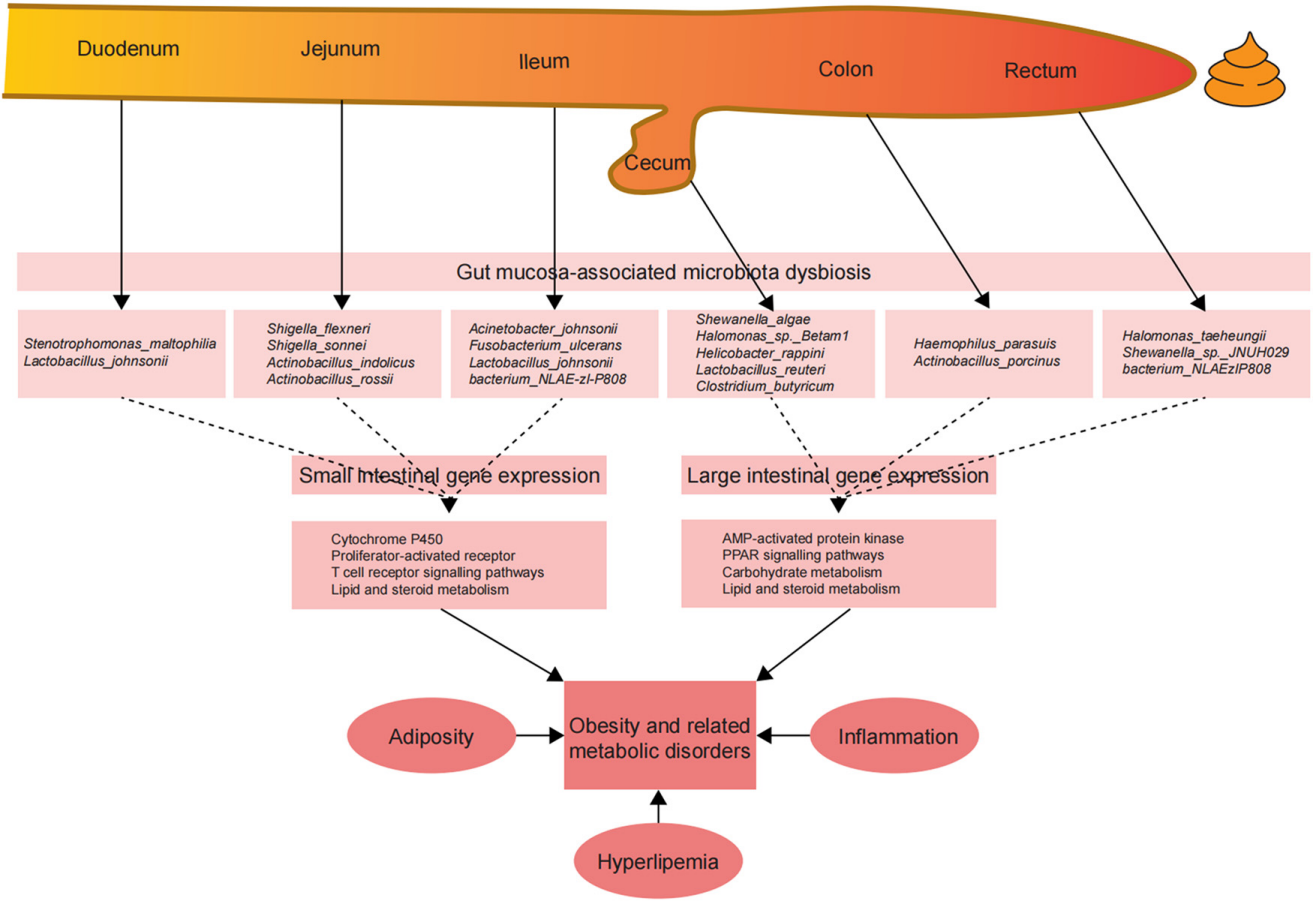
Impact of gut mucosa-associated microbiota on local and distant organs contributes to obesity development and progression. A long-term high-energy diet can cause dysbiosis of mucosa-associated microbiota across gut segments, which leads to obesity development by altering gene expression in the small and large intestine, exacerbating hyperlipemia, and triggering low-grade chronic inflammation.

Here, transcriptome analysis demonstrated that, compared to results with the ND feeding group, HED feeding altered expression of genes primarily involved in lipid metabolism and inflammatory responses in intestinal tissues. For example, pathway analysis of upregulated genes in the small intestine revealed that fat digestion and absorption, the complement system, and inflammatory pathways were enriched in the HED feeding group. The microbiota colonizing the small intestinal outer mucus layer is mainly responsible for lipid metabolism and IgA production, which are associated with metabolic disorders (30). Therefore, mucosa-associated microbiota dysbiosis may influence intestinal gene expression (7). Previous studies have demonstrated that the complement system plays a key role in maintaining host immunosurveillance and tissue homeostasis by regulating the elimination of pathogens (31). Additionally, enriched signaling pathways among upregulated genes in the small intestine, including cytochrome P450, proliferator-activated receptor (PPAR), and T-cell receptor signaling pathways, highlighted the elevated inflammatory state in the MetS model. Furthermore, large intestine transcriptome data revealed the activation of pathways involved in pyruvate and fatty acid metabolism and inflammation, including AMP-activated protein kinase and PPAR signaling, in the MetS model. Of note, both the small and large intestine showed upregulation of genes in the PPAR signaling pathway, which regulates expression of a large variety of genes involved in lipid and carbohydrate metabolism (32). The intestinal PPAR signaling pathway has also been considered an important molecular pathway for shaping gut immune responses to bacterial load and diet by regulating the recruitment and activity of various cell populations in both the innate and the adaptive immune systems (33). We previously reported decreased expression of genes related to the intestinal tight junction in the MetS model (17), leading to increased permeability of the gut barrier to endotoxins, pathogenic bacteria, and other antigens. The results of the current study suggest that mucosal microbiota dysbiosis is likely to exacerbate obesity and related metabolic disorders by regulating organic nutrient metabolism and host inflammatory pathways (Fig. 6).

In conclusion, we showed here striking differences in the mucosa-associated microbiome and transcriptome profiles along the entire length of the GI tract that were associated with obesity and related metabolic disorders in the MetS porcine model. Interactions between the mucosal microbiota and metabolic parameters were analyzed to interpret the etiological mechanism of obesity. We identified differentially expressed genes in intestinal tissues that were potentially reflective of metabolic disorders and higher inflammation in the HED feeding group. Thus, our results indicated that mucosal microbiota dysbiosis along the entire digestive tract promoted obesity, which might occur partially through amplification of systemic inflammatory responses.

## MATERIALS AND METHODS

### Study design and sample collection.

Animal maintenance and experimental treatments were conducted in accordance with the guidelines of the Animal Care and Use Committee of the Germplasm Resource Center of Chinese Experimental Minipigs. The experimental design was previously described by Xu et al. (17). Briefly, male Wuzhishan minipigs were randomly assigned to two groups: a normal diet (ND) group, which was fed a normal chow diet, and a high-energy diet (HED) group fed a high level of saturated fat and cholesterol. The HED formula comprised 3% cholesterol, 10% fat (lard), and 87% base material (48% corn, 20% wheat, 15% soybean cake, 12% rice bran, and 5% fish meal). All randomly selected individuals were euthanized at 64 months of age. We collected epithelium scrapings from GI tracts (HED group, *n* = 11; ND group, *n* = 6) that were separated into six sections of tissue: duodenum, jejunum, ileum, cecum, colon, and rectum. Additionally, the intestinal tissues (six sections) were collected from the HED and ND treatment groups. Samples were immediately snap-frozen in liquid nitrogen and stored at −80°C prior to further analysis.

### Metabolic characterization.

Total body and liver weights were measured in the two treatment groups as previously reported (17). Additionally, internal organ (heart and spleen) and visceral fat (perirenal fat, omentum, and leaf fat) weights were also measured at the endpoint of the dietary intervention study (64 months) in the two groups. Pathological examination of the liver and serum lipid profiles were also performed during month 64 of the treatment phase as previously described (17). All metabolic parameters are listed in Table 1.

### Gut mucosa-associated bacterial DNA extraction and 16S rRNA sequencing.

Gut mucosa-associated bacterial genomic DNA was extracted using a DNA extraction kit (TIANGEN Biotech, Beijing, China). For each sample, total DNA concentration and purity were measured using the NanoDrop One spectrophotometer (Thermo Fisher Scientific, Waltham, MA, USA) at 260 and 280 nm. Extracted DNA was immediately stored at −80°C. The V3-V4 hypervariable regions were amplified using universal primers with barcodes. 16S DNA libraries were recovered using a GeneJET gel extraction kit (Thermo Fisher Scientific) and quantified using the Qubit 2.0 fluorometer (Thermo Fisher Scientific). Purified DNA libraries were generated and index codes added using the NextR Ultra DNA library prep kit for Illumina (New England Biolabs [NEB], Ipswich, MA, USA) following the manufacturer’s instructions. Finally, the libraries were sequenced on an Illumina HiSeq 2500 platform with 250-bp paired-end reads using the standard protocol (Illumina, San Diego, CA, USA). Total bacterial copy numbers were quantified by qPCR as described by Bi et al. (34).

### Operational taxonomic unit clustering and microbial diversity and taxonomic analyses.

The raw reads were filtered to obtain clean reads by removing adapters pollution and low-quality reads with Trimmomatic v0.36 (35). The sample numbers retained in the working data set for further analysis were as follows: 16 duodenum (10 HED and 6 ND individuals); 9 jejunum (5 HED and 4 ND individuals); 12 ileum (7 HED and 5 ND individuals); 14 cecum (8 HED and 6 ND individuals); 10 colon (5 HED and 5 ND individuals); and 10 rectum (5 HED and 5 ND individuals). Possible chimeras were identified with UCHIME (http://www.drive5.com/usearch/manual/uchime_algo.html). Denoised sequences were clustered using USEARCH v10.0 (http://www.drive5.com/usearch/manual/uchime_algo.html), and those with similarity of ≥97% were classified as OTUs by using the mothur pipeline (36). Taxonomy was assigned with uclust in QIIME (v1.9.1; http://qiime.org/index.html) and the Silva database. Nonmetric multidimensional scaling (NMDS) analysis was conducted and NMDS plots, taxonomy, and heatmaps were visualized using R v4.0.5 (https://cran.r-project.org/).

### Gut tissue RNA extraction and transcriptome sequencing.

RNA was isolated from intestinal tissues using TRIzol reagent (Invitrogen, Shanghai, China) following the manufacturer’s standard instructions, then treated with RNase-free DNase I (TaKaRa, Shanghai, China) to remove residual genomic DNA. The RNA concentration and purity were measured using a Qubit RNA HS assay kit on a Qubit 2.0 fluorometer (Life Technologies, Grand Island, NY, USA), and rRNA was depleted using a Ribo-zero rRNA removal kit (Epicentre, Madison, WI, USA). cDNA libraries were generated with the NEBNext Ultra directional RNA library prep kit for Illumina (NEB) following the manufacturer’s protocol. The library cDNA was amplified by PCR and validated for fragment size and quantity by using the Qubit 2.0 fluorometer (Life Technologies). Finally, the libraries were sequenced on the Illumina HiSeq 2500 platform to obtain 150-bp paired-end reads.

### Transcriptome sequence analysis.

Raw sequencing reads were quality checked using FastQC v.0.11.7 (37). The index adaptors and low-quality bases (*Q* < 20) were trimmed to a minimum of 36 bp using Trimmomatic v0.36 (35). Clean reads were aligned to the Sus scrofa genome (https://www.ncbi.nlm.nih.gov/genome/?term=pig) using TopHat v2.0.10 with default parameters (38), and only reads uniquely aligned to known genes were used for further analysis. Cufflinks v2.1.1 (38) was used to assemble novel lncRNAs, annotated lncRNAs, and annotated mRNA transcripts individually using the default parameters. The coding probability was calculated for novel transcripts, which were retained if they met the following criteria: coding probability score of <0.5 in CPC v0.9-r2 (39) and CPAT (40) and identified as noncoding with CNCI v2 (41). Novel lncRNAs were defined as those that met the above criteria, were longer than 200 bp, and had at least two exons. The conservation levels for lncRNAs and mRNAs were evaluated with 8-way PhastCons scores (42). The expression levels of lncRNAs and mRNAs were calculated in fragments per kilobase of transcript per million mapped reads (FPKM) using Cuffquant v2.1.1 (38). The differentially expressed lncRNAs (DELs) and mRNAs (DEGs) between HED and ND groups were identified using the Bayes-regularized *t* test with FDR correction using Cyber-T bayesreg (43). Those with an FDR of <0.05 and |log_2_(fold change)| of >0.6 were considered statistically significant. To identify significantly enriched biological pathways among DEGs, enrichment analyses were conducted using the KEGG Pathway database (https://www.genome.jp/kegg/pathway.html).

### Statistical analyses.

Statistically significant differences between groups were evaluated using Student’s *t* test. Only microbial taxa with a relative abundance higher than 0.1% in at least 50% of samples were included in analyses. OTU abundance between the HED and ND groups was evaluated with the Mann-Whitney *U* test, with a *P* value of <0.05 considered statistically significant.

### Data availability.

The raw sequencing data generated in this study have been deposited in the NCBI SRA database with the accession numbers PRJNA831679 (gut mucosa-associated microbiota) and PRJNA833901 (intestinal transcriptome).
