# Supplementary material for: Changes in the Mucosa-Associated Microbiome and Transcriptome across Gut Segments Are Associated with Obesity in a Metabolic Syndrome Porcine Model
Source: Microbiol Spectr. 2022 Jul 7;10(4):e00717-22. doi: 10.1128/spectrum.00717-22 (PMC9430857; doi:10.1128/spectrum.00717-22)
Supplement: Supplemental file 1 — Supplemental material. Download spectrum.00717-22-s0001.pdf, PDF file, 0.6 MB [file spectrum.00717-22-s0001.pdf]

# Changes in the mucosa-associated microbiome and transcriptome across gut segments are associated with obesity in a metabolic syndrome porcine model

Song-Song Xu[a,b], Nan Wang[b], Lei Huang[a], Xiu-Ling Zhang[b,c], Shu-Tang Feng[b], Sha-Sha Liu[b], Yue Wang[b], Zhi-Guo Liu[b], Bing-Yuan Wang[b], Tian-Wen Wu[b], Yu-Lian Mu[b], Shao-Hua Hou[b], Kui Li[a,b]

[a]Shenzhen Branch, Guangdong Laboratory of Lingnan Modern Agriculture, Genome Analysis Laboratory of the Ministry of Agriculture and Rural Affairs, Agricultural Genomics Institute at Shenzhen, Chinese Academy of Agricultural Sciences, Shenzhen, China.

[b]State Key Laboratory of Animal Nutrition, Key Laboratory of Animal Genetics Breeding and Reproduction of Ministry of Agriculture and Rural Affairs of China, Institute of Animal Sciences, Chinese Academy of Agricultural Sciences, Beijing 100193, China.

[c]College of Animal Science and Technology, Nanjing Agricultural University, Nanjing 210095, China.

Song-Song Xu, Nan Wang and Lei Huang contributed equally to this work.

Corresponding authors: Kui Li, likui@caas.cn; Shao-Hua Hou, houshaohua@caas.cn

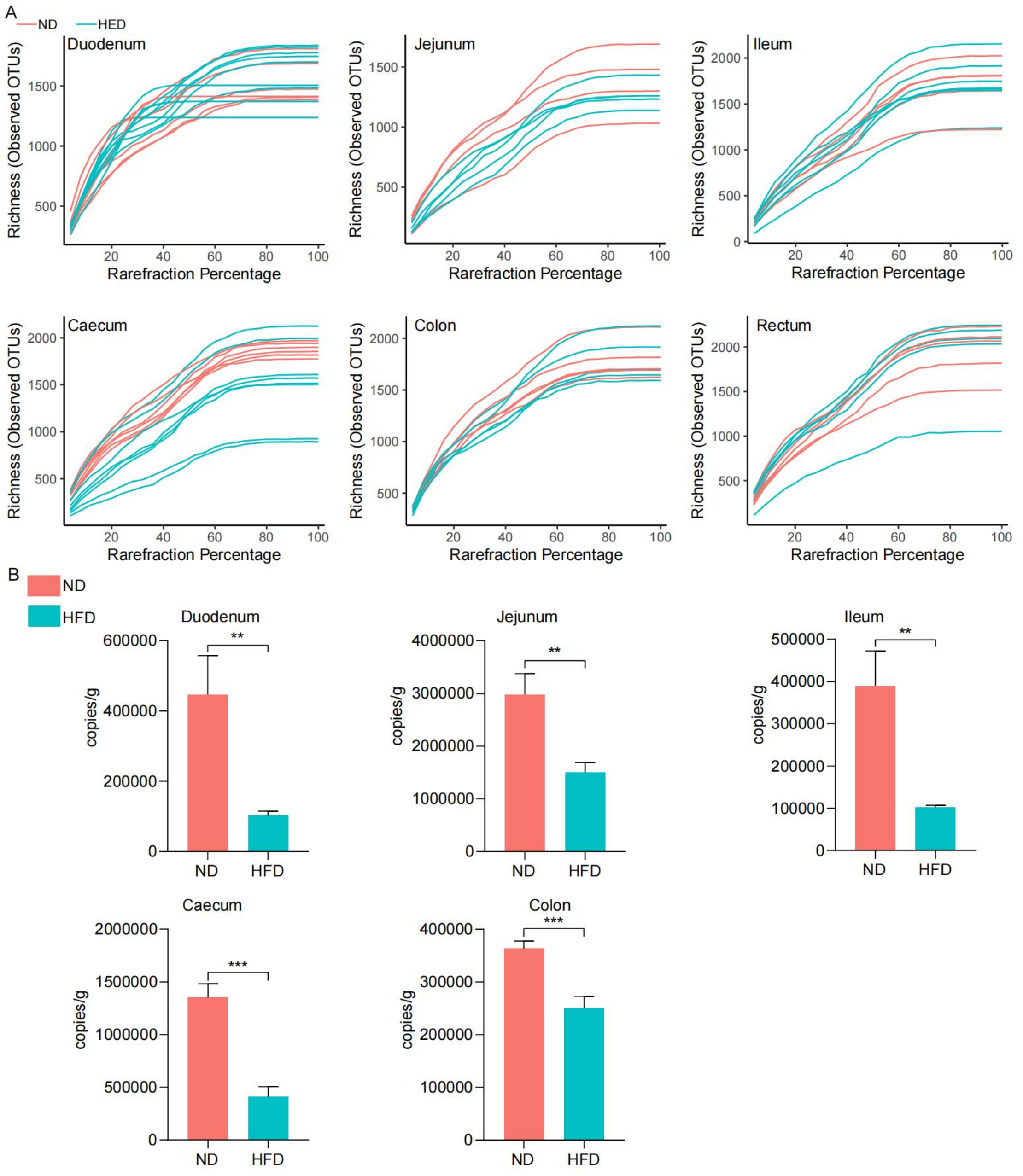


**FIG S1** (**A**) Rarefaction curves of operational taxonomic units (OTUs) for V3-V4 mapped reads from 16S rRNA genes in the GI tract. (**B**) 16S rRNA gene copy number was measured with qPCR using DNA extracted from gut mucosa-associated contents. ***p* < 0.01, ****p* < 0.001 (two-tailed Student's *t*-test).

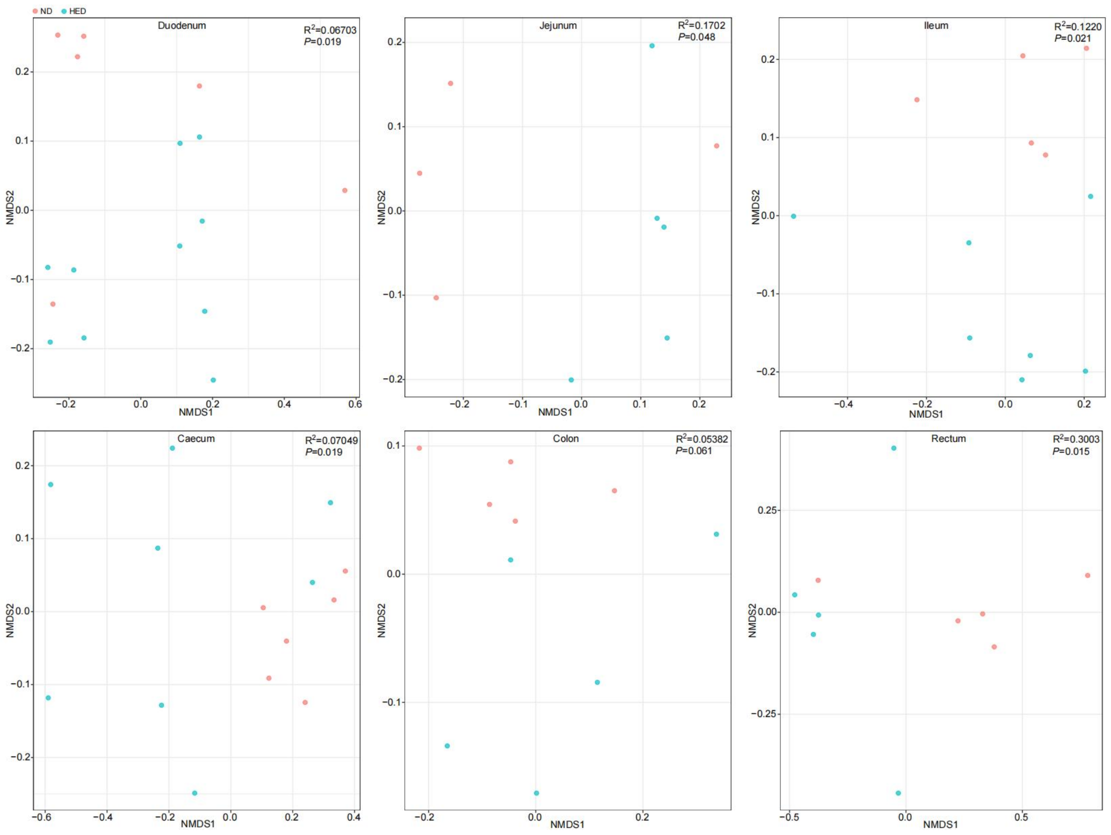


**FIG S2** Nonmetric multidimensional scaling (NMDS) analysis using Bray-Curtis dissimilarity based on OTU abundances in the GI tract.

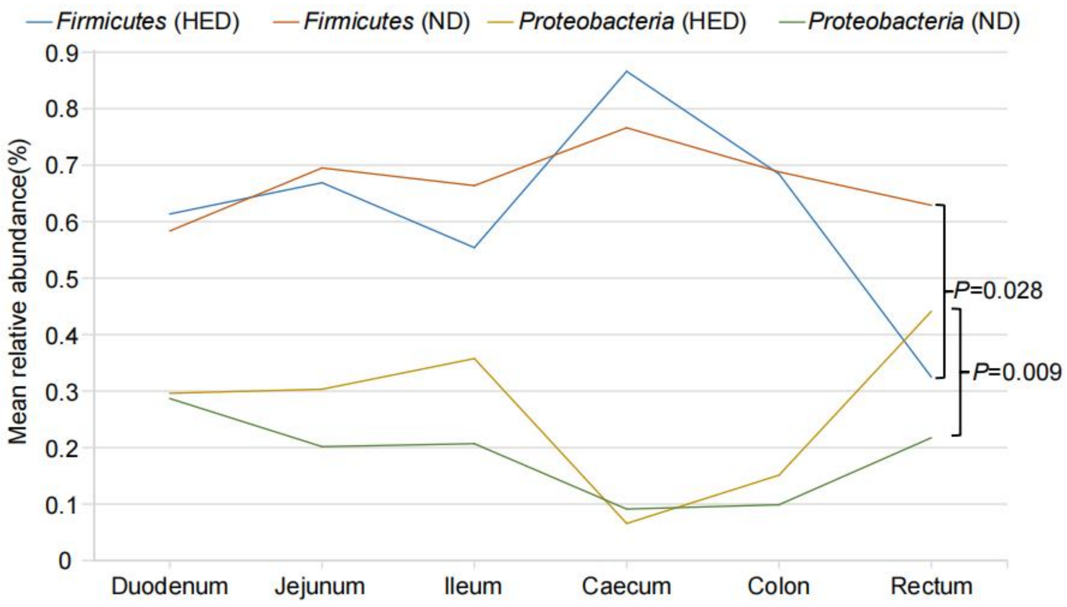


**FIG S3** Changes in relative abundance of Firmicutes and Proteobacteria along the length of the GI tract.

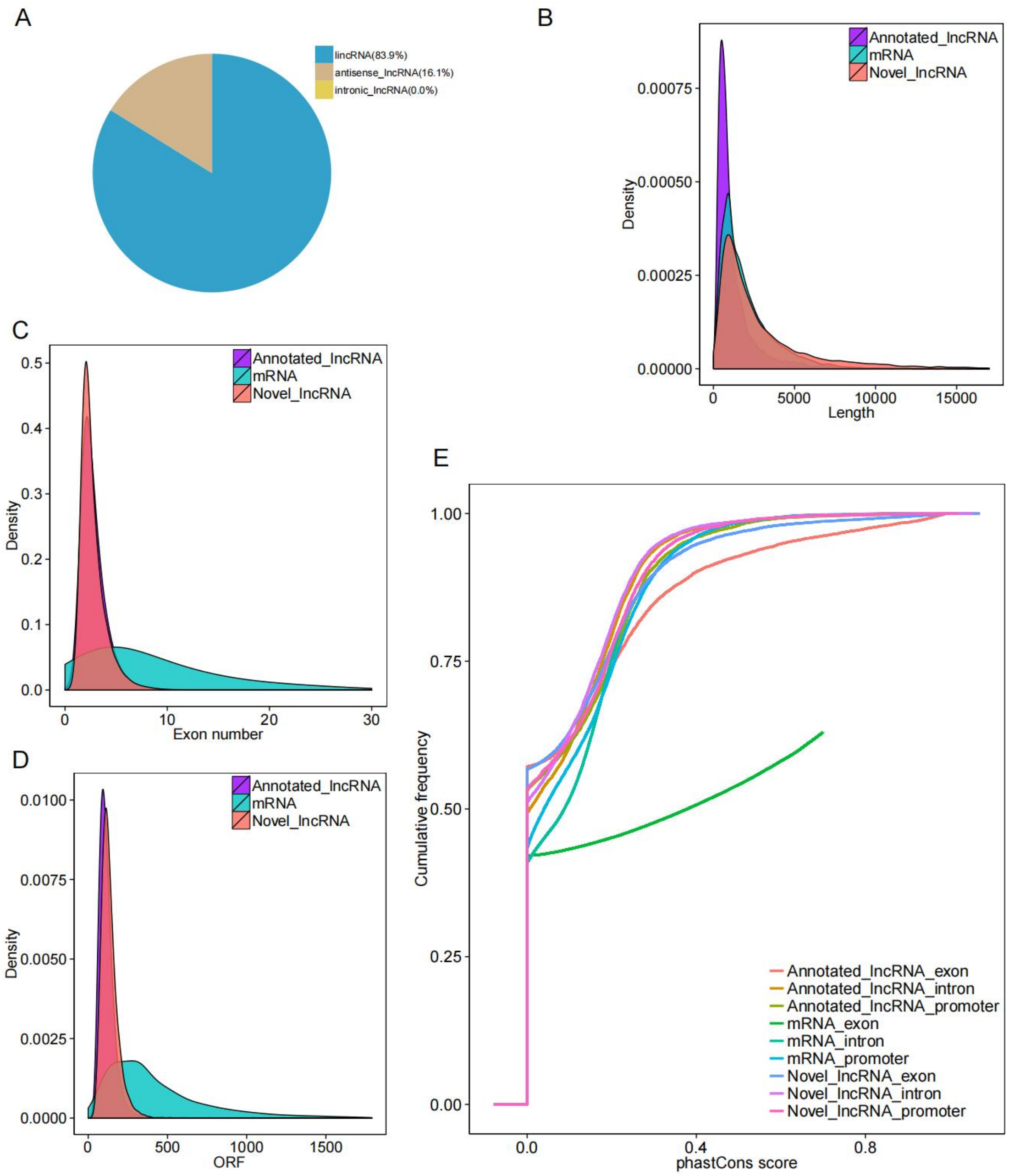


**FIG S4** Sequence characterization of lncRNAs and mRNAs. (**A**) Classification of putative lncRNAs. (**B**) Distribution of mRNA and lncRNA transcript lengths. (**C**) Distribution of mRNA and lncRNA exon numbers. (**D**) Distribution of mRNA and lncRNA open reading frame (ORF) lengths. (**E**) Cumulative distribution curve of mRNA and lncRNA conservation levels as calculated with PhastCons.
